# Patient-Controlled Epidural Levobupivacaine with or without Fentanyl for Post-Cesarean Section Pain Relief

**DOI:** 10.1155/2014/965152

**Published:** 2014-05-19

**Authors:** Shin-Yan Chen, Feng-Lin Liu, Yih-Giun Cherng, Shou-Zen Fan, Barbara L. Leighton, Hung-Chi Chang, Li-Kuei Chen

**Affiliations:** ^1^Department of Anesthesiology, Shuang Ho Hospital, Taipei Medical University, New Taipei City 10361, Taiwan; ^2^Department of Anesthesiology, School of Medicine, College of Medicine, Taipei Medical University, Taipei 10031, Taiwan; ^3^Department of Anesthesiology, National Taiwan University Hospital, National Taiwan University College of Medicine, Taipei 10002, Taiwan; ^4^Department of Anesthesiology, Washington University School of Medicine, St. Louis, MO 63110, USA; ^5^Department of Anesthesiology, National Taiwan University Hospital Hsin-Chu Branch, Hsin-Chu City 30059, Taiwan

## Abstract

*Purpose.* The purpose of this study was to compare the analgesic properties of levobupivacaine with or without fentanyl for patient-controlled epidural analgesia after Cesarean section in a randomized, double-blinded study. *Methods.* We enrolled American Society of Anesthesiologists class I/II, full-term pregnant women at National Taiwan University Hospital who received patient-controlled epidural analgesia after Cesarean section between 2009 and 2010. Eighty women were randomly assigned into two groups. In group A, the 40 subjects received drug solutions made of 0.6 mg/ml levobupivacaine plus 2 mcg/ml fentanyl, and in group B the 40 subjects received 1 mg/ml levobupivacaine. Maintenance was self-administered boluses and a continuous background infusion. *Results.* There were no significant differences in the resting and dynamic pain scales and total volume of drug used between the two groups. Patient satisfaction was good in both groups. *Conclusion.* Our study showed that pure epidural levobupivacaine can provide comparative analgesic properties to the levobupivacaine-fentanyl combination after Cesarean section. Pure levobupivacaine may serve as an alternative pain control regimen to avoid opioid-related adverse events in parturients.

## 1. Introduction

Good analgesia after Cesarean section (C/S) is important for maternal-child bonding, early ambulation, and discharge. Several studies revealed that the analgesic efficacy of neuraxial morphine was superior to that of intravenous or intramuscular opioids [1, 2]. However, adverse responses to neuraxial morphine, such as nausea or vomiting, pruritus, and dizziness, were reported to be dose related in some studies and not to be dose related in others [3–5]. One study showed that epidural local anesthesia with opioids induced better pain relief and caused less nausea/vomiting than intrathecal morphine [6].

Pure local anesthetics are not widely used for routine postoperative epidural analgesia because of the significant rate of insufficient pain relief and unacceptable incidence of motor blockade [7, 8]. However, we have been using pure epidural ropivacaine after C/S, and it offered comparable analgesic efficacy to epidural morphine without delaying the time of first ambulation [9]. Similarly, levobupivacaine has similar advantages to ropivacaine, and it has less toxicity to the central nervous system and heart and fewer motor-blocking properties than bupivacaine [10]. Based on these considerations, we posited that pure epidural levobupivacaine may also provide good analgesia after C/S.

We conducted a pilot study to determine the lowest effective concentrations of pure levobupivacaine and levobupivacaine with fentanyl to provide analgesia after C/S. The parturients were split into two groups (1 and 2). The first parturient in each group received 1.2 mg/mL levobupivacaine, and the parturients in group 1 received an additional 2 mcg/mL of fentanyl. This concentration of levobupivacaine was chosen because it was between the motor blocking minimum local anesthetic concentration and minimum local analgesic concentration of levobupivacaine based on Lacassie et al. [11] and Robinson et al.'s [12] studies, respectively. A basal infusion of 3 mL/h and a bolus of 2 mL were selected. We reduced the levobupivacaine concentration by 0.2 mg/mL for the next parturient if the previous one had lower limb numbness on the 12th hour and stopped reducing the concentration until a parturient who had a pain score > 4 (VAS; 0 = no pain at all, 10 = the worst imaginable pain) on the 12th hour or vice versa. The results of our pilot study indicated that 0.6 mg/mL levobupivacaine plus 2 mcg/mL fentanyl and 1 mg/mL of pure levobupivacaine were the lowest concentrations of the drugs required to provide effective analgesia. In this pilot study, the parturients who received these two different regimens had similar pain scores and total regimen amounts. Therefore, we hypothesized that these two regimens were “equianalgesic,” and we designed a randomized, prospective, double-blinded study to compare the analgesic properties of the two regimens.

## 2. Materials and Methods 

This research was conducted at the National Taiwan University Hospital, Taipei, Taiwan, from July 2009 to December 2010. After acquiring institutional review board approval and written informed consents, we enrolled American Society of Anesthesiologists (ASA) class I/II, full-term, healthy pregnant women who received regional anesthesia for a scheduled C/S and submitted permission for patient-controlled epidural analgesia (PCEA) in the study. Exclusion criteria were concomitant or previous intra-abdominal surgeries other than C/S, histories of intra-abdominal infection, and congenital intrauterine anomalies (i.e., septate or double uterus). All recruited women were randomly assigned to one of two groups by computer-generated randomization: group A received 0.6 mg/mL levobupivacaine plus 2 mcg/mL fentanyl and group B received 1 mg/mL levobupivacaine. In the operation room, one of two research members who prepared the study regimen inserted an epidural needle through the 3rd or 4th lumbar spinal space, turned the bevel rostrally, and threaded a catheter 5 cm into the epidural space. Anesthesia was performed with 10 mg of 0.5% hyperbaric intrathecal bupivacaine and a supplement of 2% epidural lidocaine (60 mg every 5 minutes) until a T6 sensory blockade was achieved.

The 48-hour study period began in the postanesthesia care unit (PACU) with the following PCEA settings: 2 mL for bolus, 3 mL/h for continuous infusion, a 20-minute lock out interval, and a 4-hour limit of 30 mL. Parturients received a first dose of 20 mg intravenous tenoxicam in PACU and subsequent doses every 6 hours for 48 hours. The primary outcome was the dynamic and resting VAS scores during ambulation and bed rest (on the 6th, 12th, 18th, and 24th hours); the secondary outcomes were (1) the total dose of the regimen administered on the 48th hour, (2) parturient satisfaction with the quality of pain management on the 48th hour (2 = very satisfied, 1 = satisfied, 0 = fair, −1 = dissatisfied, and −2 = very dissatisfied), and (3) parturient self-reported severity (1 = mild, 2 = moderate, and 3 = severe) of adverse events (i.e., lower limb numbness, nausea or vomiting, dizziness, pruritus, sleepiness, and urinary retention) at the 12th hour. Motor blockade was additionally rated with the Bromage score [13].  Table 1 shows the Bromage score. An acute pain service team, whose members were blinded to the regimens, recorded the primary and secondary outcome data. Parturients who used the PCEA for less than 48 hours were exempt from total volume of drug used.

### 2.1. Statistical Analysis

The number of parturients included in the study was based on a previous study by Dernedde et al. [14] and on a power calculation assuming a 20% difference with *a* = 0.05 and *b* = 0.20. Results were expressed as the mean (± standard deviation) for quantitative variables. Comparison of mean values between groups was performed using the Student's *t*-test for the VAS measurements, total drug consumptions, and adverse events. Repeated measures analysis of variance (ANOVA) was used to analyze the VAS scores within groups. Proportions were compared by the classical chi-squared test for ASA classifications, previous C/S, Bromage scores, and parturient satisfaction. Results were considered to be significant at the 5% critical level (*P* < 0.05).

## 3. Results

**Figure 1:**
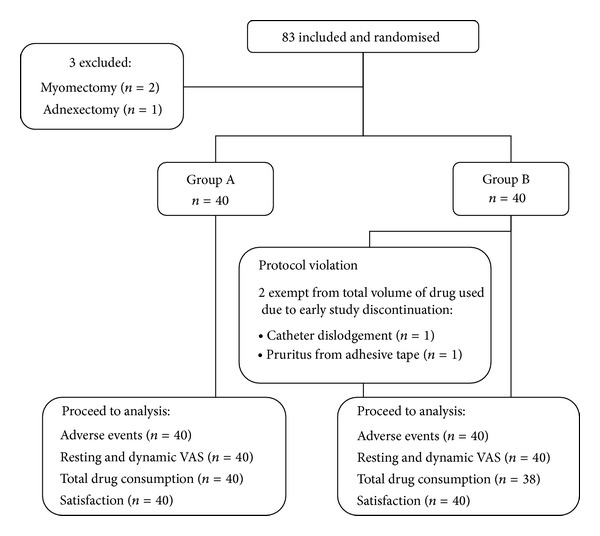
Diagram showing the flow of the patients enrolled in the study.

Figure 1 shows the flowchart of the parturients enrolled in the study. Eighty-three women were enrolled. Three were excluded because of concomitant surgeries (i.e., two myomectomies and one adnexectomy). A total of 80 women were included and randomly divided into two groups (A and B), with 40 women in each group.  Table 2 shows the baseline parturient characteristics. Two women in group B were exempted from the total volume of drug used measurements because of early discontinuation due to catheter dislodgement or intolerable pruritus associated with 3 M paper adhesive tape.

**Figure 2:**
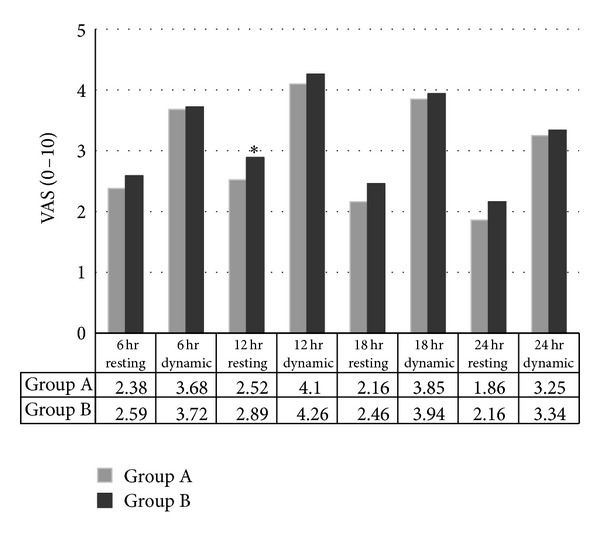
Average resting and dynamic pain scores on the 6th, 12th, 18th, and 24th hours. VAS: visual analog scale;*P = 0.03.

For the primary outcomes, the average resting and dynamic VAS scores showed no differences on the 6th, 18th, and 24th hours between the two groups (Figure 2). There was no difference in total volume of drug used: 185.6 (±25.4) mL in group A versus 184.6 (±29.7) mL in group B (*P* = 0.88). The overall satisfaction responses were as follows: 1 parturient had a score of 0, 38 parturients had a score of 1, and 1 parturient had a score of 2 in each group (Figure 4).

**Figure 3:**
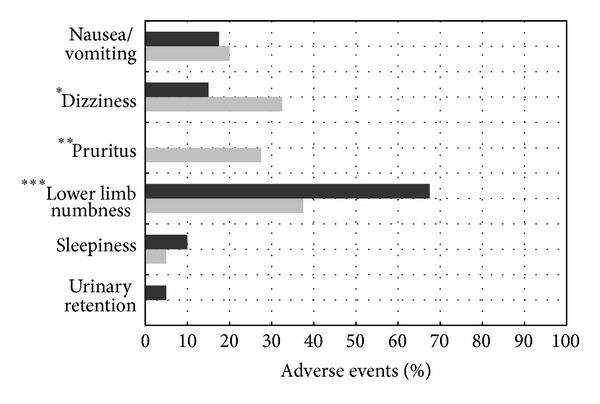
Incidence of adverse events in group A (gray column) and group B (black column). All events were mild in severity; *P = 0.019, **P = 0.0001, and ***P = 0.0045.

**Figure 4:**
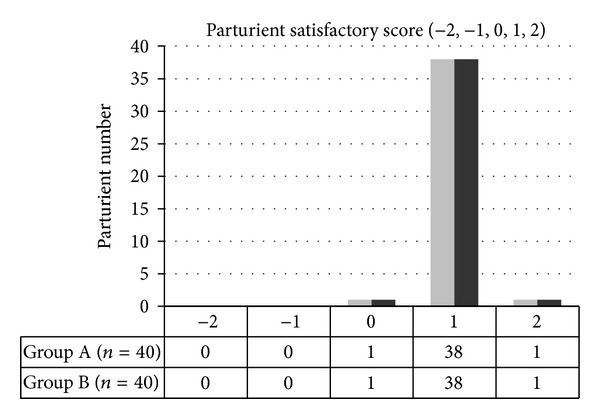
Parturient satisfactory score.

For the secondary outcomes, all subjects reported the severity of adverse events to be mild (score = 1). For group A versus group B, the incidence of nausea or vomiting was 20% versus 17.5% (*P* = 0.41), the incidence of dizziness was 32.5% versus 15% (*P* = 0.019), the incidence of pruritus was 27.5% versus 0% (*P* = 0.0001), the incidence of lower limb numbness was 37.5% versus 67.5% (*P* = 0.0045), and the incidence of urinary retention was 0% versus 5% (*P* = 0.075). The Bromage score was 4 in 37 women in group A versus 34 women in group B, and the score was 3 in three women in group A versus six women in group B (*P* = 0.29 between two groups).  Figure 3 shows the incidence of adverse events in the two groups.

There were no significant differences between parturients with previous C/S (14 in group A and 19 in group B) and parturients without previous C/S in each group in terms of the average resting and dynamic VAS scores and total drug consumptions.

## 4. Discussion 

The results of our study showed that pure levobupivacaine produced comparable analgesic properties to the levobupivacaine-fentanyl combination for post-C/S pain control. There were insignificantly higher resting and dynamic VAS scores on the 12th hour than on the 6th hour, which could be related to an incomplete regression of spinal anesthesia on the 6th hour and routine eight-hour bed rest after spinal anesthesia.

In accordance with expectations, parturients who received fentanyl suffered more from opioid-related adverse reactions, such as dizziness and pruritus, whereas those who received pure levobupivacaine experienced more local anesthetic-induced lower limb numbness. In our study, these adverse events were all mild and tolerable, and no woman was dissatisfied with the analgesic effect. Mild lower limb numbness that did not affect ambulation occurred in 67.5% of parturients in group B and in 37.5% in group A. This numbness might result from an epidural placed in a lower space than the correlated dermatome of the surgical wound (T12–L1). Visser et al. concluded that the intervertebral level is a statistically significant factor in the distribution of sensory blockade, and a low thoracic insertion was shown to cause less motor blockade than a high lumbar epidural approach [15]. Moreover, eight women (three in group A and five in group B) with lower limb numbness had symptom relief after withdrawing the catheter by 1 to 3 cm, which may have alleviated nerve root irritation [16].

As for the PCEA setting, there have been no published data on post-C/S pain control. Some studies revealed that using a low concentration and a high volume of local anesthetic provided adequate analgesia and reduced motor blockade [14, 17–21]. Some researchers concluded that PCEA with a continuous background infusion had better analgesic properties than demand-only PCEA [22–24]. In our study, we used a low concentration of levobupivacaine with a baseline infusion of 3 mL/h and a bolus of 2 mL, which was less than the concentration used in previous studies [7, 25, 26]. The satisfactory analgesia afforded in our setting might be due to the average shorter stature of the subjects in this study [27] or be due to less analgesic anticipation among Asian women.

There are several limitations in our study. First, it is difficult to define an “equianalgesic” concentration of two different regimens. Second, a parturient might not acquire adequate analgesia for uterine contractions despite several boluses, and, as a consequence, the total volume of drug used might increase while the satisfaction decreases. Third, this study did not analyze differences in the duration of C/S and total intraoperative local anesthetic use between groups and the potential impact of these factors on postoperative baseline pain levels. A further limitation is that we did not study the analgesic properties of 1.0 mg/mL levobupivacaine plus 2 mcg/mL fentanyl and pure 0.6 mg/mL levobupivacaine, since the two regimens had already been ruled out in our pilot study for the adverse effect (lower limb numbness) and insufficient analgesia, respectively.

## 5. Conclusions 

From our study, we conclude that in equivalent doses PCEA levobupivacaine with fentanyl has more dizziness and pruritus and less paraesthesia than PCEA levobupivacaine alone. Pure levobupivacaine could be an alternative regimen for parturients who have had previous negative experiences with or are concerned about opioid-related adverse events.

## Figures and Tables

**Table 1:** Bromage score.

Score	Description
4	Ability to raise extended legs
3	Inability to raise extended legs and decreased knee flexion, but full extension of feet and ankles present
2	Inability to raise legs or flex knees, but flexion of ankles present
1	Inability to raise legs, flex knees or ankles, or move toes

**Table 2:** Baseline parturient characteristics. Values are mean (SD) [95% confidence interval] or number (proportion).

	Group A (*n* = 40)	Group B (*n* = 40)	*P*
Age; years	34.1 (3.57) [32.99–35.21]	34.8 (3.4) [33.75–35.85]	0.72

Height; cm	159.8 (4.76) [158.32–161.28]	160.7 (4.7) [159.24–162.16]	0.99

Weight; kg	65.7 (9.78) [62.67–68.73]	69.1 (9.9) [66.03–72.17]	0.84

BMI; kg·m^2^	26.0 (3.2) [25.01–26.99]	26.7 (3.3) [25.68–27.72]	0.36

Gestational age; week	38.5 (1.0) [38.19–38.81]	38.6 (0.8) [38.35–38.85]	0.87

ASA class I	17 (42.5%)	16 (40%)	0.82
ASA class II	23 (57.5%)	24 (60%)	

Previous Caesarean section	14 (35%)	19 (47.5%)	0.26

BMI: body mass index; ASA: American Society of Anesthesiologists.
